# An immune and epithelial–mesenchymal transition-related risk model and immunotherapy strategy for grade II and III gliomas

**DOI:** 10.3389/fgene.2022.1070630

**Published:** 2023-01-04

**Authors:** Wei Luo, Qi Quan, Jiaxin Jiang, Roujun Peng

**Affiliations:** Department of VIP Section, State Key Laboratory of Oncology in South China, Sun Yat-sen University Cancer Center, Guangzhou, Guangzhou, China

**Keywords:** glioma, IDH1, immune, EMT, TGF-β, PD-L1

## Abstract

Grade II and III gliomas are heterogeneous and aggressive diseases. More efficient prognosis models and treatment methods are needed. This study aims to construct a new risk model and propose a new strategy for grade II and III gliomas. The data were downloaded from The Cancer Genome Atlas (TCGA), the Gene Expression Omnibus (GEO), gene set enrichment analysis (GSEA), and the EMTome website for analysis. The Human Cell Landscape website and the Genomics of Drug Sensitivity in Cancer website were used for single-cell analysis and drug susceptibility analysis. Gene set enrichment analysis, gene function enrichment analysis, univariate and multivariate Cox regression analyses, Pearson’s correlation analysis, log-rank test, Kaplan–Meier survival analysis, and ROC analysis were performed. We constructed an immune-related prognostic model associated with the isocitrate dehydrogenase 1 (IDH1) mutation status. By analyzing the immune microenvironment of patients with different risk scores, we found that high-risk patients were more likely to have an inflammatory immune microenvironment and a higher programmed death ligand-1 (PD-L1) expression level. Epithelial–mesenchymal transition (EMT)-related gene sets were significantly enriched in the high-risk group, and the epithelial–mesenchymal transition phenotype was associated with a decrease in CD8^+^ T cells and an increase in M2 macrophages. Transforming growth factor-β (TGF-β) signaling was the most important signaling in inducing epithelial–mesenchymal transition, and TGFB1/TGFBR1 was correlated with an increase in CD8^+^ T cytopenia and M2 macrophages. Survival analysis showed that simultaneous low expression of TGFBR1 and PD-L1 had better survival results. Through single-cell analysis, we found that TGFB1 is closely related to microglia and macrophages, especially M2 macrophages. Finally, we discussed the sensitivity of TGFB1 inhibitors in gliomas using cell line susceptibility data. These results demonstrated a potential immunotherapy strategy in combination with the TGFB1/TGFBR1 inhibitor and PD-1/PD-L1 inhibitor for grade II and III gliomas.

## 1 Introduction

Grade II and III gliomas are the most common primary brain tumors and proved to be with substantial heterogeneity in terms of pathological features and clinical outcomes (Louis et al., 2021). In order to distinguish the different pathological features of patients and develop individualized treatment strategies, glioma-related biomarkers have been identified. The IDH1 was reported to mutate frequently in gliomas (Yan et al., 2009). It had been proved that gliomas with the IDH1 mutation were more sensitive to chemotherapy and radiotherapy, resulting in a better prognosis (Sanson et al., 2009). Other biomarkers, for example, codeletion of 1p and 19q (1p/19q) (Smith et al., 2000), Capicua (CIC) transcriptional repressor mutation (Gleize et al., 2015), loss of chromosome 9p, mutation of phosphatidylinositol-4,5-bisphosphate 3-kinase catalytic subunit alpha (PIK3CA) and phosphoinositide-3-kinase regulatory subunit 1 (PIK3R1) (Draaisma et al., 2015), and deletion of cyclin-dependent kinase inhibitor 2 A (CDKN2A) (Reis et al., 2015), were confirmed to have prognostic value and important for rational selection of surgery, radiotherapy, and chemotherapy treatment. However, over 50% of grade II and III gliomas eventually develop into highly aggressive gliomas, indicating the need for a more efficient prognosis model and treatment methods (Zhu et al., 2021).

In the past decades, there has been little progress in the treatment of gliomas. Although immunotherapy successfully promoted the treatment results of other cancer types and was the major research direction for gliomas, limited progression had been made in immunotherapy treatment of gliomas (Yang et al., 2022). In CheckMate-498, a randomized clinical trial, nivolumab combined with bevacizumab and nivolumab combined with chemoradiotherapy in newly diagnosed glioma patients with O^6^-methylguanine DNA methyltransferase (MGMT) promoter unmethylation were both ineffective (Reardon et al., 2020). Comparing with IDH-wild gliomas, the IDH-mutant gliomas have significantly low tumor-infiltrating lymphocytes and PD-L1 expression (Berghoff et al., 2017). Numerous studies had demonstrated that gliomas were infiltrated by immune cells that made up to 30% of a tumor’s mass (Kaminska et al., 2021). Such extensive accumulation of innate immune cells in gliomas might be misleading as these events did not reflect the effective anti-tumor immunity. This phenomenon enlightened us that tumor immune infiltration in gliomas might be accompanied by other pathological processes that promote tumor progression.

As mentioned previously, the construction of a new risk model and improving the efficacy of immunotherapy are essential for the treatment of gliomas. In this study, we attempted to construct an immune-related risk model and proposed a feasible strategy for improving the immunotherapy efficacy for gliomas.

## 2 Materials and methods

### 2.1 RNA sequencing data

The IDH1 somatic mutation status for 500 samples, gene expression data for 525 samples, and the corresponding clinical datasheets for 515 samples were obtained from TCGA website (https://portal.gdc.cancer.gov/). Among these grade II and III glioma samples, 493 samples with RNA sequencing data and IDH1 mutation information were subjected to subsequent analyses. Log2 (x+1) normalization was performed for all gene expression data. Rows and columns with more than 50% missing values were removed. The study report fully met TCGA publication requirements.

### 2.2 Microarray data

The gene expression profile matrix files from GSE107850 (including 195 samples) and GSE43388 (including 43 samples, 15 from GSE43388-GPL570 and 28 from GSE43388-GPL8542) were downloaded from the GEO database (https://www.ncbi.nlm.nih.gov/geo/). We used the R package inSilicoMerging [DOD: 10.1186/1471–2105–13–335] to merge the datasets. Also, we used empirical Bayes methods (Johnson et al., 2007) to remove batch effects.

### 2.3 Construction and validation of the risk model

GSEA was performed to determine how the immunological pathways and corresponding immune genes differ between IDH1-wild (IDH1-wt) (*n* = 117) and IDH1 mutation (IDH1-mut) (*n* = 376) samples in TCGA cohort. An annotated gene set file (c7. immunesigdb.v7.4. symbols.gmt; downloaded from the Molecular Signatures Database) was selected for use as the reference gene set. The expression profiles of the top 50 genes expressed in the IDH1-wt and IDH1-mut groups were analyzed *via* univariate Cox regression analysis. In this analysis, genes were regarded as significant at *p* < 0.05. For the significant genes, least absolute shrinkage and selection operator (LASSO) Cox analysis was performed by using the glmnet R package. Then, the nine candidate genes were analyzed by multivariate Cox regression analysis based on progression-free survival (PFS). Finally, two independent prognostic factors for PFS were analyzed by multivariate Cox regression analysis based on overall survival (OS) to construct the risk model $\left(Risk\;Score=\underset{i}{\sum }\left.Coefficient\;of\left(i\right)*Expressionofgene(i)\right)\right.$ . The log-rank test and Kaplan–Meier survival analysis were used to assess the predictive ability of the prognostic model. The maxstat R package was applied to determine the best cutoff value, and then patients were classified into low-risk and high-risk groups. We performed ROC analysis using the R package pROC.

### 2.4 Correlation of immunophenotype with the risk model

Using the deconvo_CIBERSOR method of the IOBR R package, the immune cell infiltration score was calculated. The R package ESTIMATE was used to calculate the immune scores for each patient based on gene expression. Pearson’s correlation coefficients for risk scores and immune infiltration were calculated. The rank sum test was used to compare differences in immune cells and immune-related molecules between high-risk and low-risk groups. *p* < 0.05 was considered significant.

### 2.5 Functional enrichment analysis

The STRING website (https://cn.string-db.org/) was used to construct a 32-gene functional protein association network. We used the Kyoto Encyclopedia of Genes and Genomes Application Programming Interface (KEGG API) to obtain genetic annotations for the latest KEGG pathway. Enrichment analysis was performed using the R package clusterProfiler.

### 2.6 Epithelial–mesenchymal transition (EMT) gene set-related analysis

The 61 EMT gene sets were downloaded from the EMTome website (http://emtome.org/) (Vasaikar et al., 2021). GSEA was performed between the high-risk and low-risk groups. The enrichment scores of the EMT gene sets were analyzed by univariate Cox analysis through the EMTome website. The gene set with the highest prognostic significance and the highest GSEA enrichment score was selected for gene set variation analysis (GSVA) to obtain the enrichment scores of samples. Pearson’s correlation coefficients for enrichment scores, risk scores, immune cells, and immune-related molecules were calculated. The log-rank test and Kaplan–Meier survival analysis were applied to assess the predictive ability of the enrichment score. By reviewing the literature, we identified five EMT phenotype-related signaling pathways and downloaded corresponding gene sets from the GSEA website (http://www.gsea-msigdb.org/), namely, REACTOME_SIGNALING_BY_TGFB_FAMILY_MEMBERS, GOBP_CANONICAL_WNT_SIGNALING_PATHWAY, BIOCARTA_RAS_PATHWAY, GOBP_NOTCH_SIGNALING_PATHWAY, and GOBP_PHOSPHATIDYLINOSITOL_3_KINASE_SIGNALING (Dongre and Weinberg, 2019). We divided the samples into two groups according to the enrichment score, with 263 samples in the high-enrichment score group and 262 samples in the low-enrichment score group, and performed GSEA to obtain the most significant enrichment signaling pathway.

### 2.7 Effects of transforming growth factor-β (TGF-β)-associated molecules, PD-L1 (CD274), and CTLA-4 on immune cell infiltration and prognosis

Pearson’s correlation coefficients for enrichment scores, TGFB1, TGFBR1, TGFB2, and TGFB3 were calculated, and the correlation matrix was plotted. Multivariate Cox regression analysis was performed on TGFB1, TGFBR1, TGFB2, TGFB3, PD-L1, and cytotoxic T-lymphocyte-associated protein 4 (CTLA-4). For TGFB1, TGFBR1, CD274, CTLA-4, and immune cells, we calculated the Pearson’s correlation coefficient and plotted the correlation scatterplot. The log-rank test and Kaplan–Meier survival analysis were used to assess the predictive ability of TGFBR1 and PD-L1. The maxstat R package was applied to determine the best cutoff value, and then patients were classified into the low-expression group and the high-expression group. The samples were then divided into four groups (TGFBR1-H+CD274-L, TGFBR1-H+CD274-H, TGFBR1-L+CD274-L, and TGFBR1-l+CD274-H) for survival analysis, and the differences in immune cell infiltration between groups were compared by the rank sum test, and violin charts were plotted.

### 2.8 Single-cell analysis was performed to determine cell localization of TGF-β signaling-related molecules

This analysis was conducted through the Human Cell Landscape website (https://db.cngb.org/HCL/index.html) (Han et al., 2020). Platform creators analyzed >700,000 single cells from >50 human tissues (2–4 replicates per tissue in general) and cultures. Through the brain section of the Gallery module, we can acquire the single-cell data matrix and analysis results related to brain tissue.

### 2.9 Anti-TGFB1 drug susceptibility analysis

We adopted the Genomics of Drug Sensitivity in Cancer website (https://www.cancerrxgene.org/) and the EMTome website (http://emtome.org/) for drug sensitivity analysis. The two websites provided drug susceptibility data on the TGFB1 inhibitor LY2109761 in different cell lines, as well as online analysis tools. Using online tools from both websites, we performed drug susceptibility analysis.

### 2.10 Data processing platform

All the data processing was performed on Sangerbox (http://vip.sangerbox.com/home.html), a powerful platform based on R (Shen et al., 2022), including RNA sequencing data normalization, merging of datasets, removing batch effects, gene set enrichment analysis, gene set variation analysis, gene function enrichment analysis, univariate and multivariate Cox regression analyses, LASSO-Cox regression analysis, Pearson’s correlation analysis, log-rank test, Kaplan–Meier survival analysis, ROC analysis, the immune cell infiltration score and immune infiltration score calculation, and KEGG enrichment analysis.

## 3 Results

### 3.1 Construction and validation of a risk model associated with IDH1 and immune status

**FIGURE 1:**
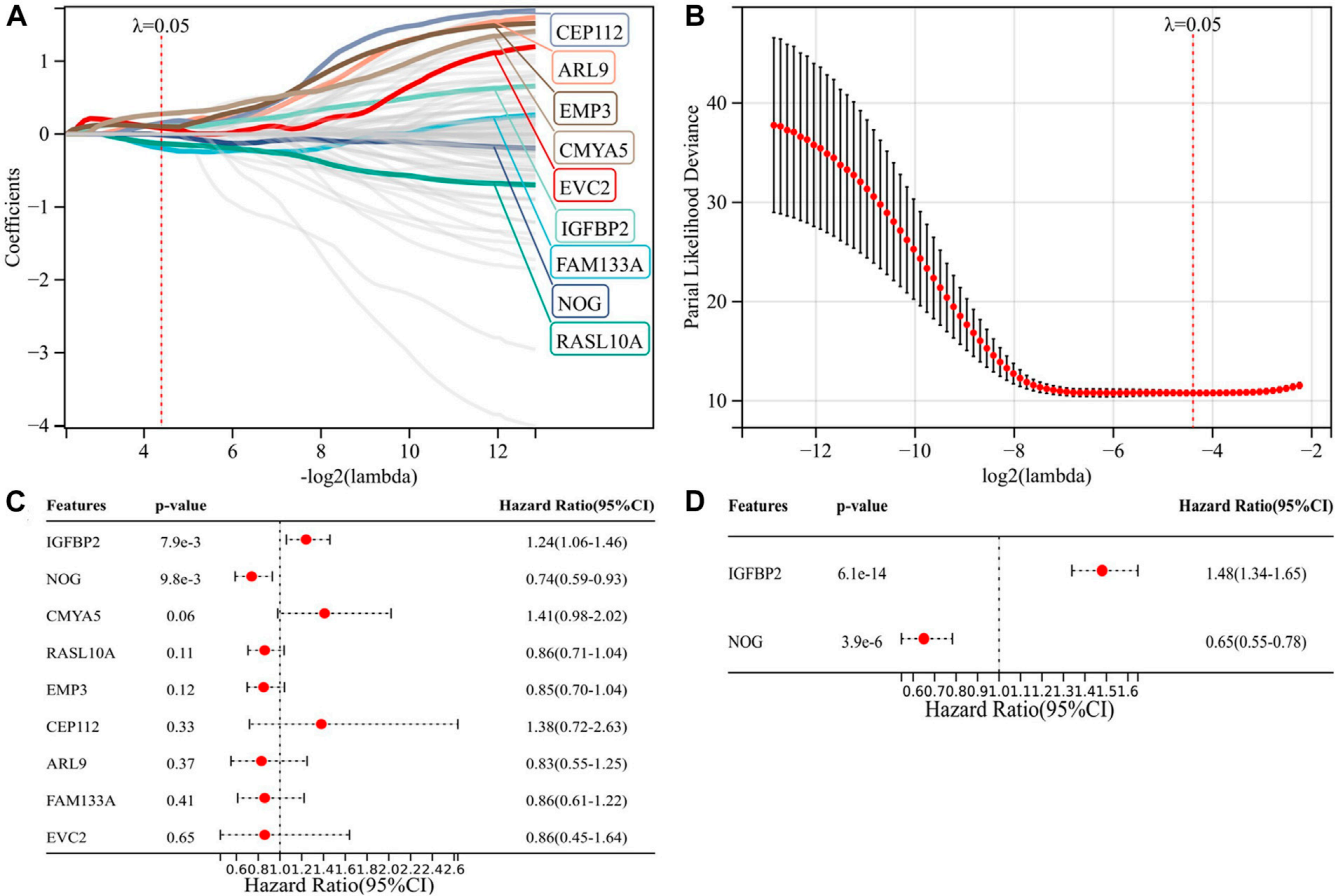
Construction of a risk model associated with IDH1 and immune status. **(A–B)** Nine candidate genes for risk modeling by LASSO Cox regression analysis; **(C)** multivariate analysis of nine genes on PFS; and **(D)** multivariate analysis of two genes on OS.

We applied GSEA between IDH1-wt (*n* = 117) and IDH1-mut (*n* = 376) groups using c7. immunesigdb.v7.4. symbols.gmt as the reference gene set. All 3919 immune-related gene sets with FDR < 0.05 were enriched in the IDH1-wt group, suggesting that the IDH1-wt group was more correlated with immune response than the IDH1-mut group. The top 50 genes enriched in the two groups were used for univariate Cox regression analysis. The results showed that all 100 genes were significantly associated with prognosis (Figure S1). These 100 genes were then analyzed by LASSO Cox, and nine candidate genes were obtained for risk modeling (Figures 1A–B). In order to better predict PFS, we performed multivariate analysis on PFS for nine genes and obtained two genes with independent prognostic value for PFS, NOG, and IGFBP2 (Figure 1C). Through multivariate analysis of NOG and IGFBP2 in OS, we obtained the expression coefficients of these two genes and constructed a risk model (Figure 1D). Risk score = -0.4253 * expression of NOG + 0.3954 *expression of IGFBP2.

**FIGURE 2:**
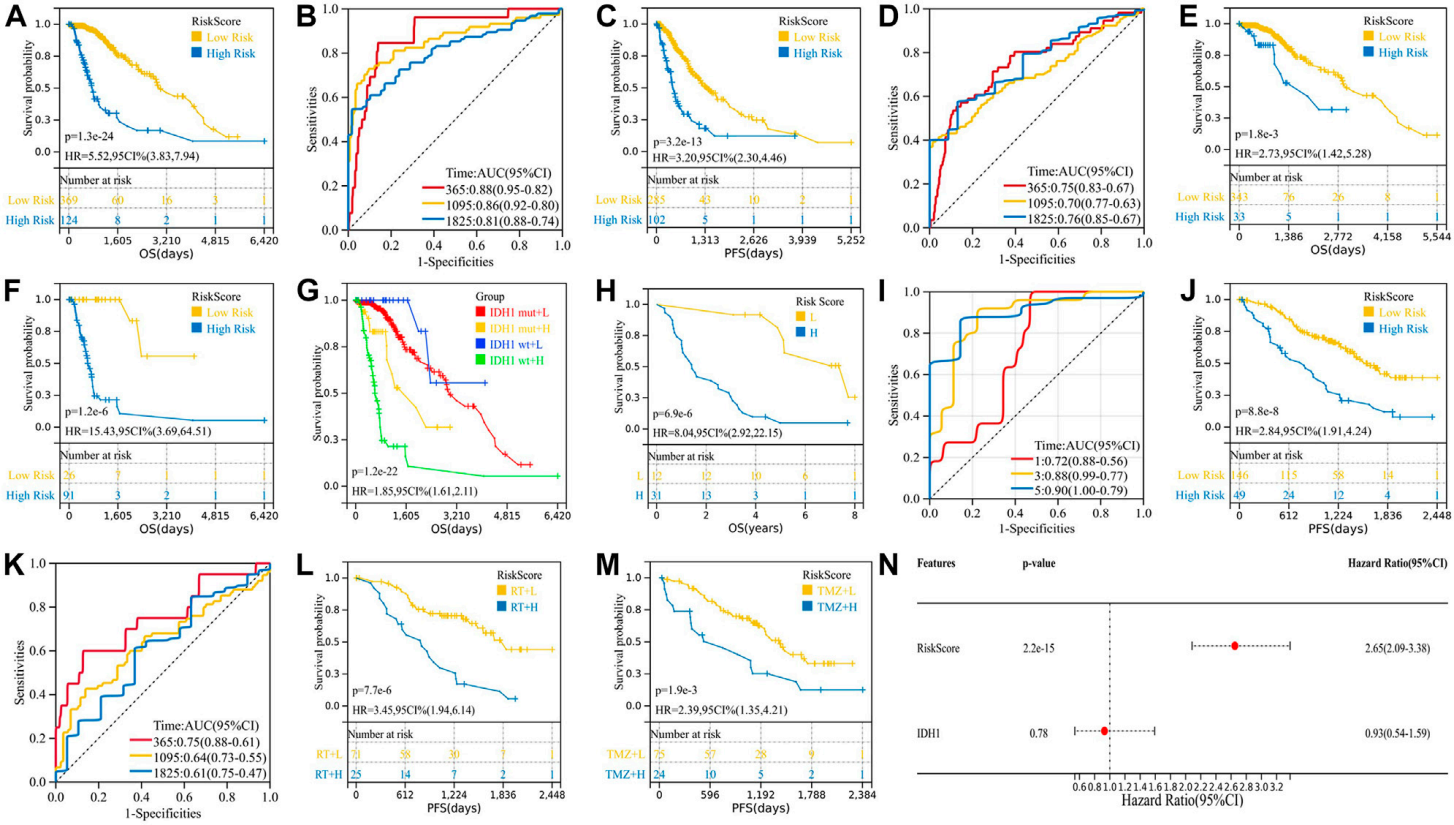
Validation of the two-gene risk model. **(A)** K-M survival curve for OS of TCGA cohort; **(B)** ROC curve for OS of TCGA cohort; **(C)** K-M survival curve for PFS of TCGA cohort; **(D)** ROC curve for PFS of TCGA cohort; **(E)** high-risk patients in the IDH1-mut group have shorter OS; **(F)** high-risk patients in the IDH1-wt group have shorter OS; **(G)** K-M curve combining the IDH1 mutation status and risk score; **(H–I)** validation of the two-gene risk model in GSE43388; **(J–M)** validation of the two-gene risk model in GSE107850; and **(N)** multivariate analysis of IDH1 and risk score.

We calculated the risk scores of all 493 TCGA samples and performed survival analysis, plotting K-M curves and ROC curves. The results showed that the risk model was a good predictor of patients’ OS (*p* = 1.3e-24, HR = 5.52) and PFS (*p* = 3.2e-13, HR = 3.20) (Figures 2A–D). Clinically, the IDH1 mutation status was used to determine patient prognosis, and we explored whether our risk model could further stratify the IDH1 mutation status. In both the IDH1-mut and IDH1-wt groups, a high-risk score indicates a worse prognosis (Figures 2E–F), whereas the IDH1 status in the low-risk group does not affect prognosis (Figure 2G). Multivariate analysis of IDH1 and risk score suggested that risk score is an independent prognostic factor relative to IDH1 (Figure 2N).

GSE107850 (including 195 samples) and GSE43388 (including 43 samples) were used to validate the risk prediction capabilities of risk models. Among them, the study endpoint of the GSE107850 sample was PFS after radiotherapy (RT) and temozolomide (TMZ) treatment, and the study endpoint of the GSE43388 sample was OS, so we used these two sets of sample data to verify the predictive efficacy of risk models on PFS and OS, respectively. Furthermore, we used the GSE107850 dataset to analyze the predictive power of risk models for response to radiotherapy and chemotherapy treatment. As shown in Figures 2H–M, the risk model can predict not only the patient’s OS (*p* = 3.2e-6, HR = 7.89) and PFS (*p* = 8.8e-8, HR = 2.84) but also the patient’s response to radiation (*p* = 7.7e-6, HR = 3.45) and chemotherapy (*p* = 1.9e-3, HR = 2.39).

### 3.2 High-risk patients are more likely to have a “hot” immune microenvironment

**FIGURE 3:**
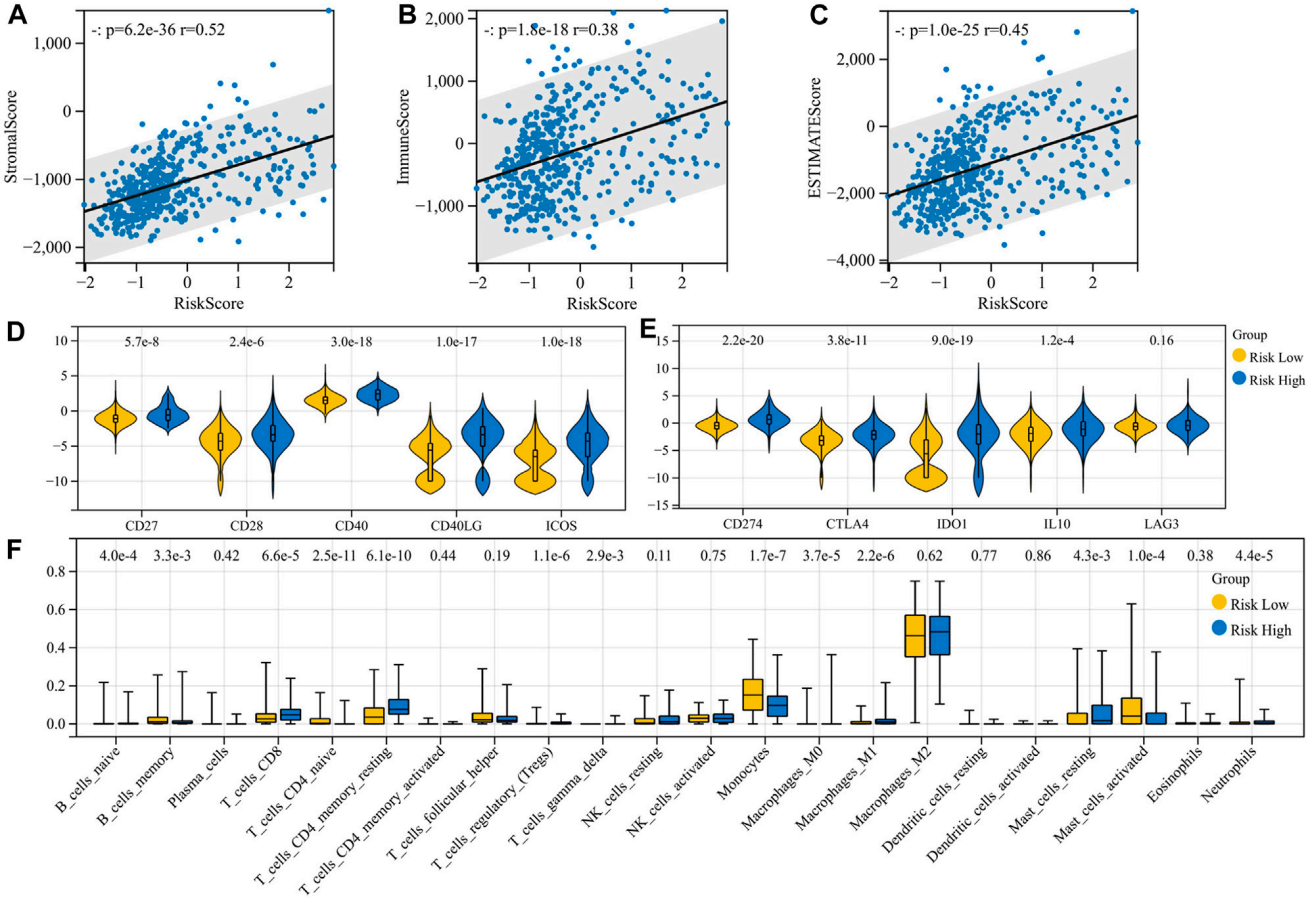
Immunocorrelation analysis of the risk model. **(A–C)** Correlation of the risk model with immune infiltration scores; **(D–E)** correlation of the risk model with the expression of immune molecules; and **(F)** correlation of the risk model with immune cell infiltration.

We analyze the correlation of the risk model with immune infiltration scores, immune cell infiltration, and expression of immune molecules. There is a significant positive correlation between immune risk scores and immune infiltration scores, including stromal score (*p* = 6.2e-36, r = 0.52), immune score (*p* = 1.8e-18, r = 0.38), and ESTIMATE score (*p* = 1.0e-25, r = 0.45) (Figures 3A–C). The high-risk group had higher expression of immunosuppressive molecules (CD274, CTLA-4, IDO1, and IL10) and also had higher expression of immunostimulant molecules (CD27, CD28, CD40, CD40LG, and ICOS) (Figures 3D–E). Regulatory T cells (Tregs) were elevated in the high-risk group (*p* = 1.1e-6). However, B naïve cells (*p* = 4.0e-4), mast resting cells (*p* = 4.3e-3), CD4+ T memory resting cells (*p* = 6.1e-10), CD8^+^ T cells (*p* = 6.6e-5), neutrophils (*p* = 4.4e-5), and M0 (*p* = 3.7e-5) and M1 (*p* = 2.2e-6) macrophages were also elevated in the high-risk group. Risk stratification did not appear to affect M2 macrophage infiltration results (*p* = 0.62) (Figure 3F). From these results, a higher level of inflammation coexisted with a higher level of immunosuppression, but unfortunately, although this immunosuppression was accompanied by increased PD-L1 expression, it could not be reversed by PD-L1 inhibitors and transformed into clinical benefit. We hypothesized that along with this immunosuppressive process, there were other malignant phenotypes that promoted tumor progression.

### 3.3 EMT-related signaling pathways are closely related to the risk model

**FIGURE 4:**
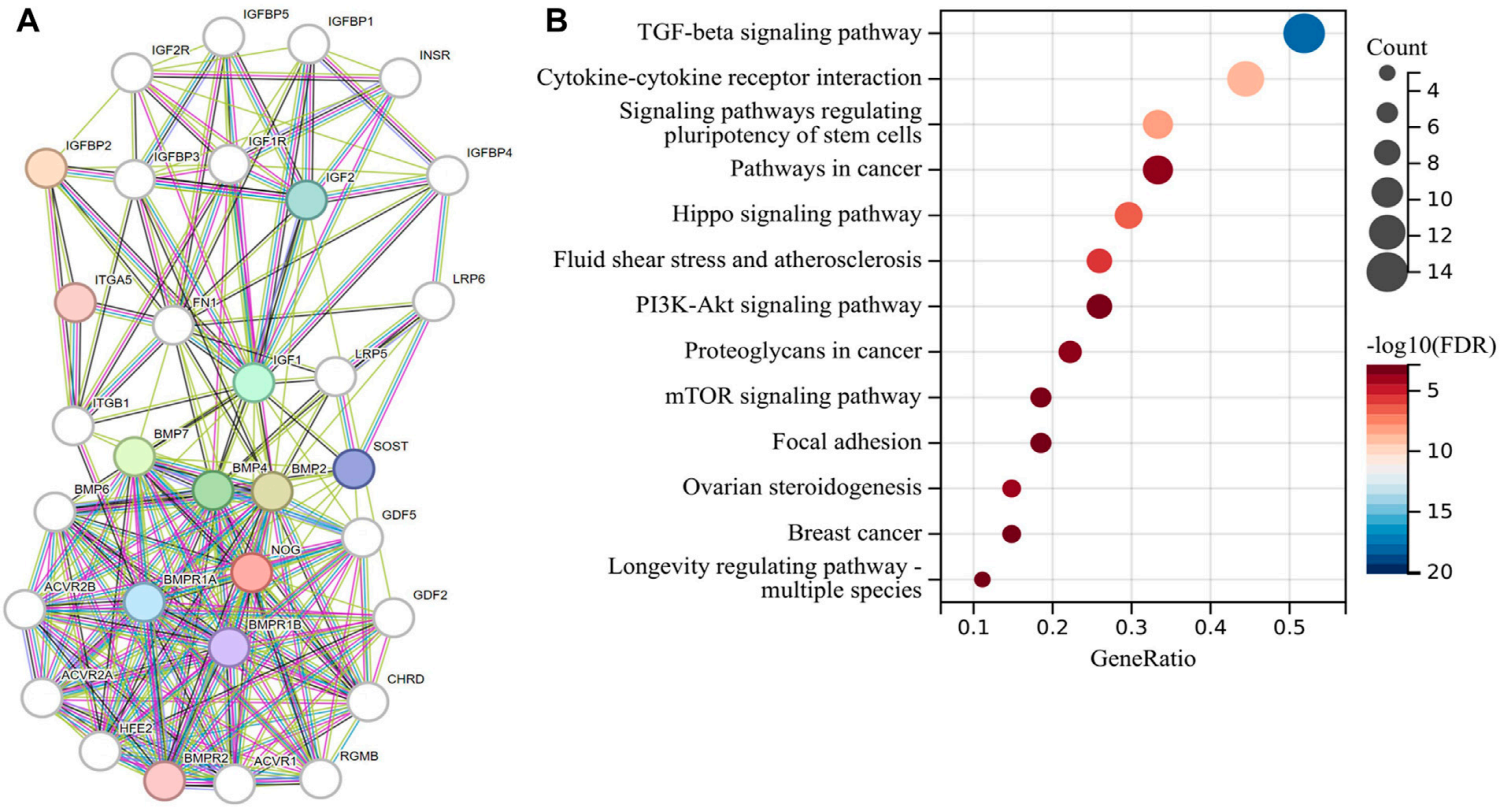
Risk model-related gene function enrichment analysis. **(A)** Gene regulatory network of 32 genes related to the risk model and **(B)** KEGG enrichment analysis of this regulatory network.

In order to explore the signaling pathways related to the risk model, we first used the STRING website to construct a gene regulatory network of 32 genes for the risk model (Figure 4A), and then we performed KEGG enrichment analysis on this regulatory network (Figure 4B). Some signaling pathways that are closely related to EMT had been enriched, including TGF-β signaling pathway (Hao et al., 2019), signaling pathways regulating pluripotency of stem cells (Mani et al., 2008; Shibue and Weinberg, 2017), Hippo signaling pathway (Cordenonsi et al., 2011), PI3K-Akt signaling pathway, and mTOR signaling pathway (Song et al., 2014). This suggested that the EMT phenotype may be also formed at the same time as the tumor formed an inflammatory immune microenvironment.

### 3.4 EMT phenotypes are significantly associated with M2 macrophages and are regulated by the TGF-β signaling pathway

**FIGURE 5:**
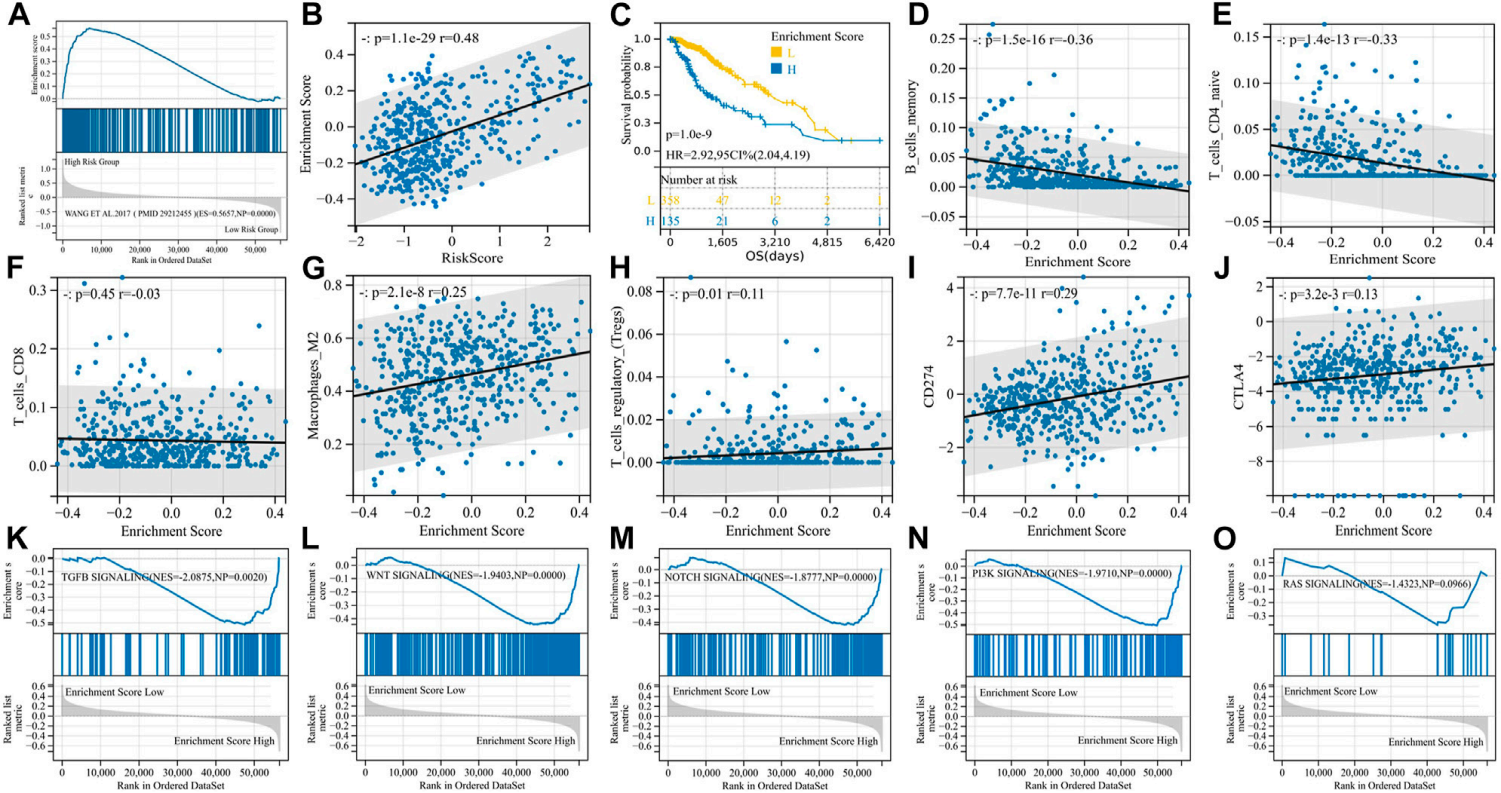
EMT phenotypes are significantly associated with M2 macrophages and are regulated by the TGF-β signaling pathway. **(A)** GSEA enrichment curve for the PMID29212455: Wang_et_al. 2017 gene set; **(B)** enrichment score and risk score are significantly positively correlated; **(C)** K-M curve for the enrichment score; **(D–J)** correlation analysis of enrichment scores and immunity; and **(K–O)** GSEA enrichment curve for EMT-related signaling pathways.

The EMTome website provided 61 gene sets that are associated with the EMT phenotype, and we downloaded all of them for GSEA between the two risk groups. At the same time, we used the online tool of the EMTome website to perform univariate Cox regression analysis of each gene set enrichment score. The top gene sets are listed in Table 1; Table 2. PMID29212455: Wang_et_al. 2017 was the gene set with the most significant prognostic value and highest normalized enrichment score (NES), and we plotted the GSEA enrichment curve for this gene set (Figure 5A).

**TABLE 1:** Univariate Cox regression analysis for EMT gene sets by the EMTome website.

Signature	Cox coefficient	Hazard ratio	Log-rank *p*-value
PMID29212455: Wang_et_al. 2017	1.38	4 (2.6–6.1)	8.00E-12
PMID26061747: Huang_et_al. 2015	1.22	3.4 (2.2–5.1)	5.40E-10
PMID29700419: Liang_et_al. 2018	1.16	3.2 (2.1–4.8)	3.90E-09
PMID29440769: Chae_et_al. 2018	1.15	3.1 (2.1–4.7)	6.00E-09
PMID20215510: Choi_et_al. 2010	1.13	3.1 (2.1–4.6)	4.80E-09
PMID24004852: Cieslik_et_al. 2013	1.13	3.1 (2.1–4.7)	1.40E-08
PMID19666588: Creighton_et_al. 2009	1.11	3 (2–4.5)	1.50E-08
PMID26088755: Kim_et_al. 2015	1.09	3 (2–4.4)	1.20E-08
PMID26771021: MsigDB_v7.0	1.1	3 (2–4.4)	1.30E-08

Note: By the online tool of the EMTome website, univariate Cox regression analysis was applied to determine the effects of different EMT phenotypes and gene set enrichment scores on the survival of grade II and III gliomas. *p* < 0.05 was considered statistically significant.

**TABLE 2:** GSEA rank list for EMT gene sets.

Term	ES	NES	p-value	FDR	FWER
PMID29212455: Wang_et_al. 2017	0.5657	2.3413	0.0000	0.001	0.001
PMID25744723: Schliekelman_et_al. 2015	0.4556	2.2449	0.0000	0.0005	0.001
PMID30728376: Soo Min_et_al. 2019	0.5228	2.2231	0.0000	0.0003	0.001
PMID24510113: Reka_et_al. 2014	0.7297	2.2166	0.0000	0.0003	0.001
PMID29346386: Hollern_et_al. 2018	0.5984	2.2083	0.0000	0.0002	0.001
PMID20713713: Taube_et_al. 2010	0.6191	2.1953	0.0000	0.0001	0.001
PMID23734191: Zarkoob_et_al. 2013	0.6186	2.1953	0.0000	0.0002	0.001
PMID19340593: Joyce_et_al. 2009	0.6535	2.184	0.0000	0.0001	0.001
PMID25214461: Tuan_et_al. 2014	0.594	2.175	0.0000	0.0001	0.001
PMID29700419: Liang_et_al. 2018	0.6864	2.1655	0.0000	0.0003	0.002

Note: EMT gene sets were acquired from the EMTome website, and GSEA was performed between the high-risk and low-risk groups. FDR <0.05 was considered significantly different in gene set enrichment between the two groups.

We then performed GSVA with the PMID29212455: Wang_et_al. 2017 gene set and obtained an enrichment score for each sample. Enrichment scores had a significant positive correlation with risk scores (*p* = 1.1e-29, r = 0.48) (Figure 5B), and high enrichment scores indicated shorter survival (*p* = 1.0e-9, HR = 2.92) (Figure 5C). The enrichment score was positively correlated with immunosuppressive cells and immunosuppressive molecules, for example, Tregs, M2 macrophages, PD-L1, and CTLA-4, but negatively correlated with B memory cells and CD4+ T cells, suggesting the immunosuppressive properties of the EMT phenotype (Figures 5D–J). In particular, unlike risk scores, EMT enrichment scores were positively correlated with M2 macrophage infiltration (*p* = 2.1e-8, r = 0.25) but not with CD8^+^ T-cell infiltration (*p* = 0.45, r = -0.03).

In order to determine the main signaling pathways that induce EMT phenotypes, we reviewed the relevant literature, identified five candidate signaling pathways, downloaded the gene sets of each pathway through the GSEA website, and then divided the samples into two groups according to the EMT enrichment score for GSEA. The TGF-β signaling pathway, WNT signaling pathway, Notch signaling pathway, and PI3K signaling pathway were all significantly enriched in the high-enrichment score group, with the TGF-β signaling pathway having the largest NES (|NES| = 2.0875), and we believed that TGF-β signaling played an important role in inducing EMT phenotypes in gliomas (Figures 5K−O).

### 3.5 Compared with PD-L1 and CTLA-4, TGFB1/TGFBR1 is much associated with the immunosuppressive microenvironment

**FIGURE 6:**
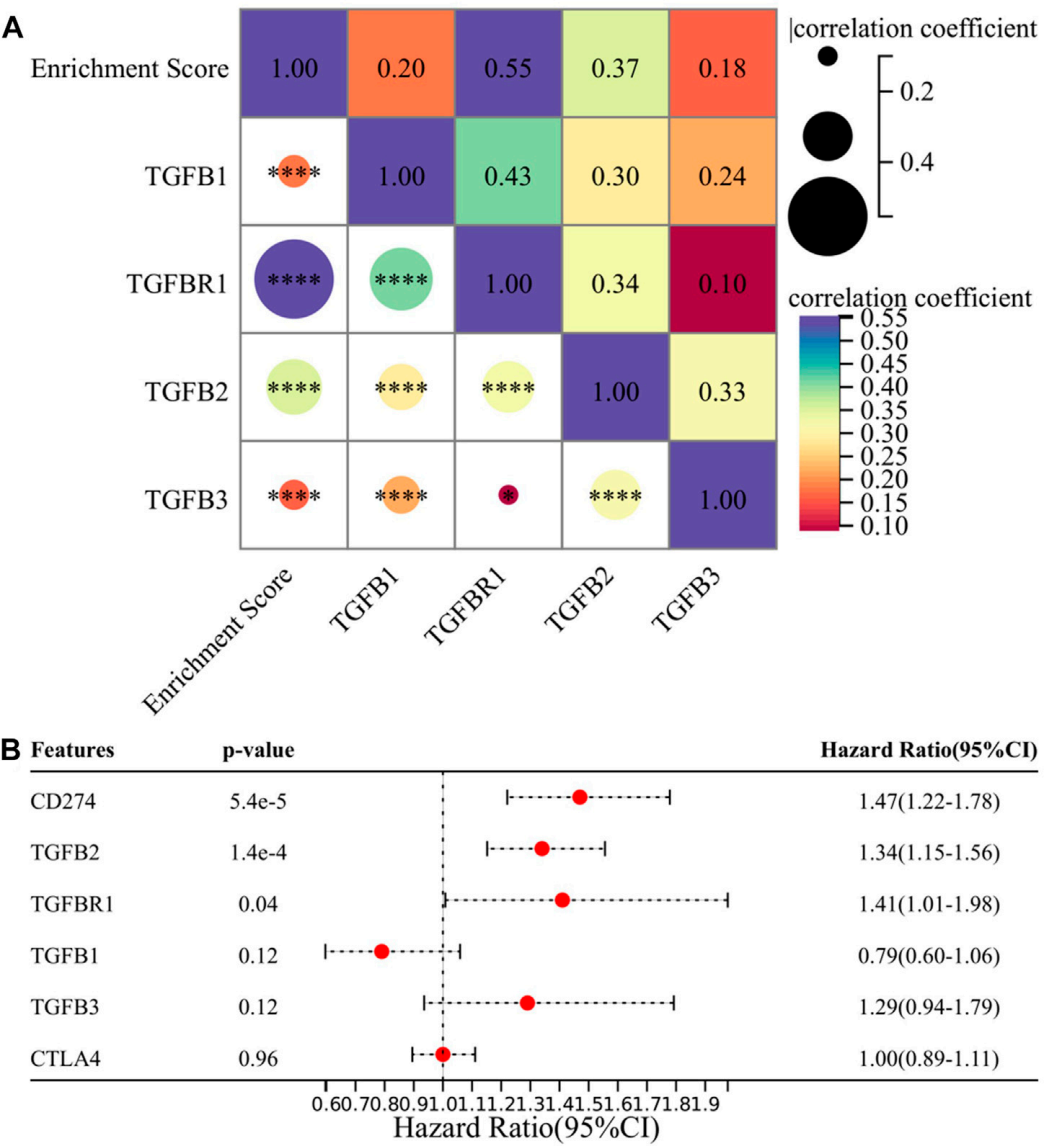
TGFB1/TGFBR1 may be a suitable target in the treatment of gliomas. **(A)** Correlation between TGFB1/TGFBR1, TGFB2, TGFB3, and enrichment score and **(B)** multivariate survival analysis of TGFB1/TGFBR1, TGFB2, TGFB3, PD-L1, and CTLA-4.

In this section, we explored specific molecules of the TGF-β signaling pathway, and we selected TGFB1, TGFBR1, TGFB2, and TGFB3 as research subjects because the drugs targeting these molecules are currently in the clinical research stage (Tauriello et al., 2022), and exploring them will make our research conclusions more likely to guide clinical practice. First, we analyzed the correlation between four candidate molecules and EMT enrichment scores, and the results showed that all four molecules were significantly correlated with EMT enrichment scores, among which TGFBR1 had the highest correlation (*p* < 0.001, r = 0.55) (Figure 6A). We then performed multivariate Cox regression analysis on four candidate molecules together with PD-L1 and CTLA-4, and the results suggested that PD-L1, TGFB2, and TGFBR1 were independent prognostic factors (Figure 6B). Based on the previous conclusions, we selected TGFB1/TGFBR1 for subsequent analysis.

**FIGURE 7:**
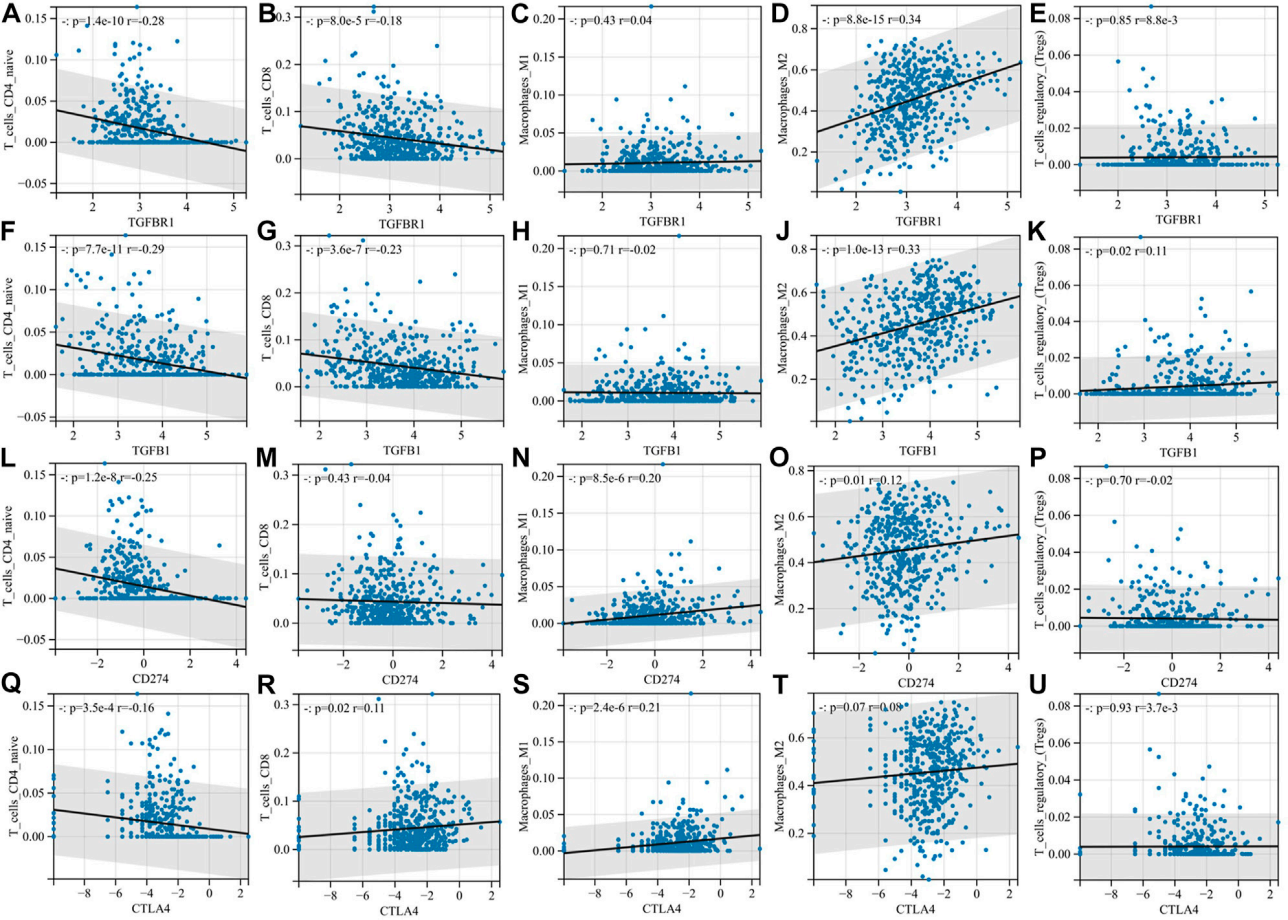
Compared with PD-L1 and CTLA-4, TGFB1/TGFBR1 is more associated with the immunosuppressive microenvironment. **(A–E)** Correlation of TGFBR1 with immune cell infiltration; **(F–K)** correlation of TGFB1 with immune cell infiltration; **(L–P)** correlation of PD-L1 with immune cell infiltration; and **(Q–U)** correlation of CTLA-4 with immune cell infiltration.

Next, we focused on the correlation between TGFB1/TGFBR1, PD-L1, CTLA-4, and immune cell infiltration. With the increase in TGFB1/TGFBR1 expression, the infiltration of CD4^+^ T naïve cells and CD8^+^ T cells (*p* = 8.0e-5, r = -0.18; *p* = 3.6e-7, r = -0.23) decreased significantly, while the infiltration of M2 macrophages (*p* = 8.8e-15, r = -0.34; *p* = 1.0e-13, r = 0.33) and Tregs increased significantly (Figures 7A–K). Although CD4^+^ T naïve cell infiltration was decreased with PD-L1 expression, there was no significant change in CD8^+^ T cells and Tregs. In addition, M1 macrophage infiltration was also increased (*p* = 8.5e-6, r = 0.20), while M2 macrophage infiltration was relatively low (*p* = 0.01, r = 0.12), suggesting that M0 macrophages were more likely polarized toward M1 macrophages (Figures 7L–P). As for CTLA-4, CD4^+^ T naïve cell infiltration also decreased, while CD8^+^ T cell (*p* = 0.02, r = 0.11) and M1 macrophage (*p* = 2.4e-6, r = 0.21) infiltration increased, and M2 macrophages and Tregs showed no significant changes (Figures 7Q–U). Compared with PD-L1 and CTLA-4, TGFB1/TGFBR1 was tightly associated with a decrease in CD8^+^ T cells and an increase in M2 macrophages and Tregs infiltration, suggesting that TGFB1/TGFBR1 may have more powerful immunosuppressive properties.

### 3.6 Simultaneous blocking of TGFB1/TGFBR1 and PD-L1 might significantly improve survival

**FIGURE 8:**
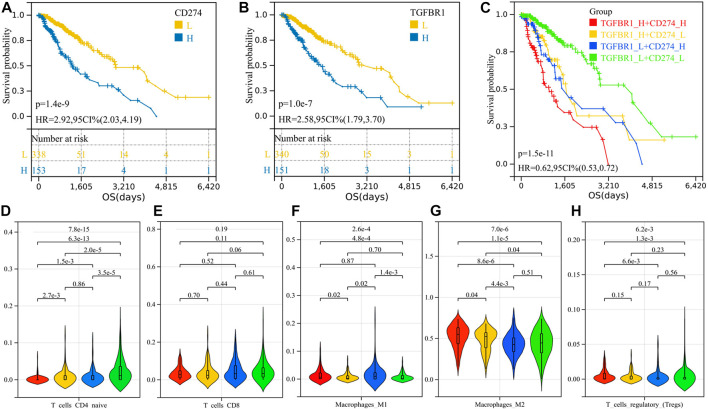
Survival analysis of PD-L1 and TGFBR1 and comparison of immune cell infiltration between groups. **(A)** K-M curve for PD-L1; **(B)** K-M curve for TGFBR1; **(C)** K-M curve for patients with different TGFBR1 and PD-L1 expression levels; and **(D–H)** immune cell infiltration between groups.

We calculated the optimal cutoff value for TGFBR1 and PD-L1 using the R package maxstat (maximally selected rank statistics with several *p*-value approximations, version: 0.7-25). We then grouped the samples according to cutoff values and performed survival analysis. Patients with higher PD-L1 and TGFBR1 expression had shorter survival (*p* = 1.4e-9, HR = 2.92; *p* = 1.0e-7, HR = 2.58) (Figures 8A–B). Patients with both high expression of TGFBR1 and PD-L1 had the worst prognosis, and those with low expression of both TGFBR1 and PD-L1 had the best prognosis, while those with high expression of one of the two molecules had a moderate prognosis (Figure 8C; Supplementary Table S1). The group with the best prognosis tended to have more CD4^+^ T naïve cells and CD8^+^ T-cell infiltration (although no statistically significant difference was achieved) and less M2 macrophage and Tregs infiltration (Figures 8D–H). These results suggested that simultaneous blocking of TGFB1/TGFBR1 and PD-L1 is more likely to confer a survival benefit than blocking PD-L1 alone.

### 3.7 TGFB1 is closely related to microglia and macrophages in brain tissues

**FIGURE 9:**
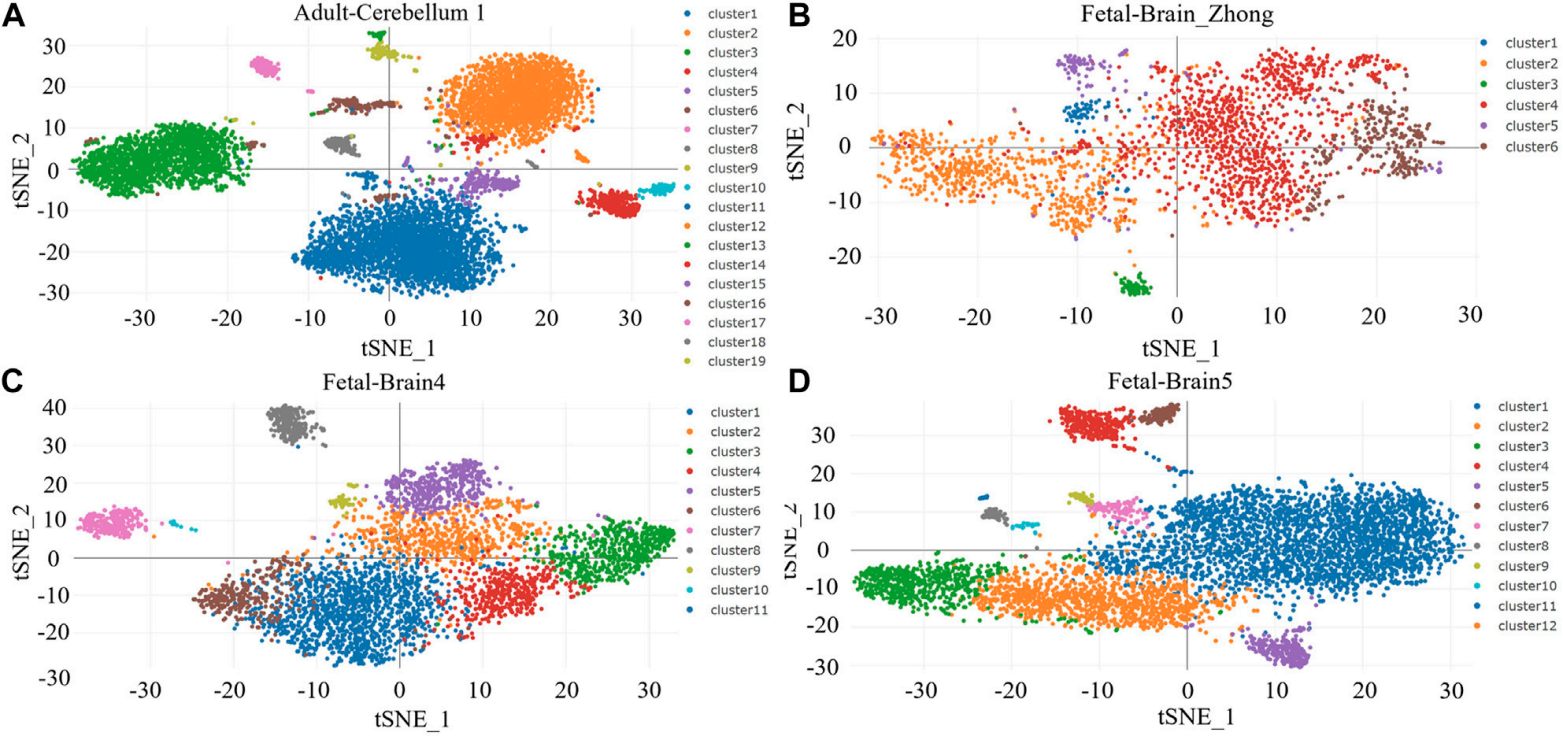
**(A–D)** Clustering analysis for brain tissues.

We analyzed the cellular localization of TGF-β-related molecules in brain tissue through Human Cell Landscape, a single-cell analysis website. In four single-cell samples of brain tissue, we found that TGFB1 and TGFBI (transforming growth factor-beta induced, a protein induced by TGFB1) were mainly expressed in microglia and macrophages, especially M2 macrophages. The results of the clustering analysis with marker genes are shown in Figures 9A–D and Table 3.

**TABLE 3:** TGFB1 and TGFBI are the top markers for microglia/macrophage in the brain.

Cluster	Annotation	Gene	*p*_value	avg_diff	pct.1	pct.2
Adult-Cerebellum1_Cluster16	Macrophage	TGFBI	5.82E-63	1.8518	0.367	0.011
Fetal-Brain_Zhong_Cluster3	Microglia	TGFB1	1.46E-48	1.1991	0.284	0.013
Fetal-Brain4_Cluster10	Macrophage	TGFBI	1.33E-15	0.8792	0.158	0.005
Fetal-Brain5_Cluster11	M2 macrophage	TGFBI	4.94E-30	1.6913	0.348	0.016
Fetal-Brain5_Cluster6	Fibroblast	TGFBI	6.48E-91	1.5493	0.254	0.011

Note: Single-cell differential gene expression analysis for four brain tissues was performed on the Human Cell Landscape website; *p* < 0.05 was considered statistically significant.

### 3.8 Gliomas may be one of the tumors most sensitive to the TGFB1 inhibitor

**FIGURE 10:**
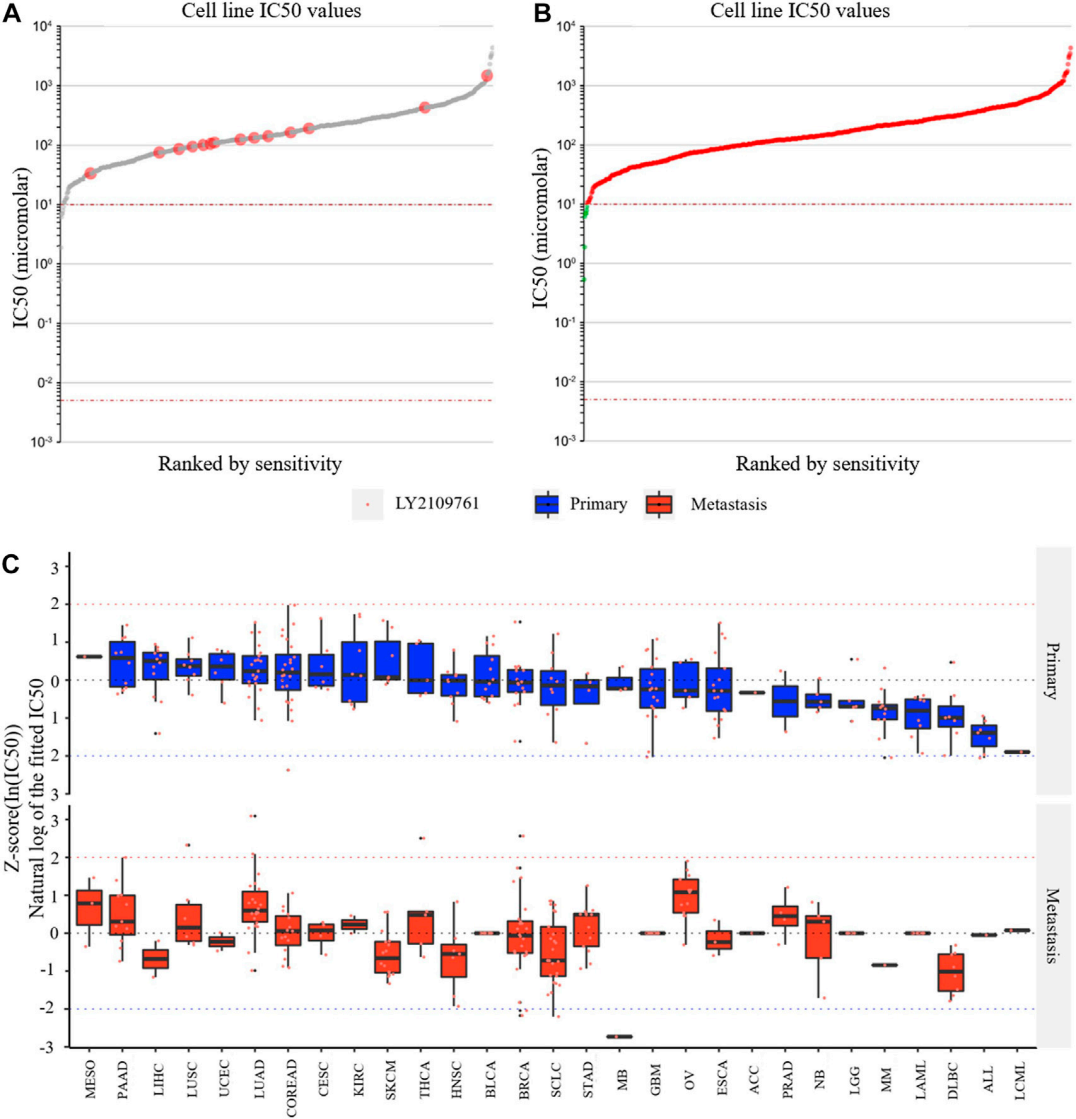
Drug susceptibility analysis of the TGFB1 inhibitor. **(A)** IC_50_ values of LY2109761 in different LGG cell lines; **(B)** IC_50_ values of LY2109761 in all available tumor cell lines; and **(C)** LGG ranked sixth in drug sensitivity among 29 tumor species.

LY2109761 is an inhibitor of TGFB1 and is widely used in *in vitro* studies of various tumors. Through the Genomics of Drug Sensitivity in Cancer website and the EMTome website, we analyzed the susceptibility results of LY2109761 in various tumors. Figure 10A shows the IC_50_ values of LY2109761 in different LGG cell lines, and the IC_50_ values of LY2109761 in all available tumor cell lines are shown in Figure 10B. After standardizing the susceptibility data of all tumor cell lines, we found that LGG ranked sixth in drug sensitivity among 29 tumor species (Figure 10C). This suggested that TGFB1/TGFBR1 inhibitors may be sensitive in gliomas.

## 4 Discussion

Previous studies had demonstrated that the IDH1 mutation status has a significant effect on the immune microenvironment of diffuse glioma, and IDH1 wild-type patients generally have higher lymphocyte infiltration and PD-L1 expression (Yan et al., 2009; Berghoff et al., 2017). Also, the IDH1 mutation status has a significant correlation with prognosis (Sanson et al., 2009). Therefore, we can utilize these features of IDH1 to construct a risk model related to the inflammatory immune microenvironment and survival. Through GSEA, we found that the 3,919 immune-related gene sets with significant differences between the two groups were enriched in the IDH1-wild group, suggesting that the IDH1-wild type is more related to immune response, which is consistent with the conclusions mentioned previously. According to the gene rank list of GSEA, top 50 genes in each group were selected for univariate analysis, and the results showed that these 100 genes had significant prognostic value. By performing LASSO Cox and multivariate Cox regression analyses on 100 genes, we constructed a two-gene risk model consisting of IGFBP2 and NOG.

IGFBP2 is a pleiotropic oncogene and plays a role in the occurrence and development of a variety of tumors (Brouwer-Visser and Huang, 2015). It has been confirmed that IGFBP2 can induce tumor epithelial–mesenchymal transformation and metastasis through the NF-κB signaling pathway (Gao et al., 2016). NOG is a natural inhibitor of bone morphogenetic protein (BMP), especially BMP2 and BMP4, which are the members of the TGF-β family. BMPs suppressed the tumorigenic function of human glioma-initiating cells by inducing cell differentiation, cell cycle arrest, and apoptosis (Bao et al., 2013). Several reports have shown that BMP4 is expressed in low-grade gliomas and that it serves as a favorable prognostic marker in gliomas (Bao et al., 2013; Nayak et al., 2020; Zhou et al., 2020). In addition, BMP4 was able to abolish cancer stem cell populations in human cancers, including malignant gliomas (Piccirillo et al., 2006; Piccirillo and Vescovi, 2006). Interestingly, as an inhibitor of BMPs, the expression of NOG and BMP2/4 was positively correlated. The high-risk group had lower levels of BMPs, consistent with the studies mentioned previously. We verified that the risk model has good prognostic value in TCGA cohort and two GEO cohorts and can further stratify the IDH1-mut and IDH1-wt groups. The multivariate Cox regression analyses further confirmed the independent prognostic value of the risk model. Thus, this model might be used as a risk indicator in clinical practice.

To further investigate the relationship between the risk model and the immune microenvironment, we analyzed the correlation between immune infiltration scores, immune-related molecules, and immune cell infiltration with immune-related prognostic models. The stromal score, immune score, and ESTIMATE score were positively correlated with the risk score. The high-risk group had higher expression of immunosuppressive molecules and also had higher expression of immunostimulant molecules. Tregs were elevated in the high-risk group. However, B naïve cells, mast resting cells, CD4+ T memory resting cells, CD8+ T cells, neutrophils, and M0 and M1 macrophages were also elevated in the high-risk group. Similar conclusions had been reached in other people’s studies. Berghoff et al. (2017) found significantly higher levels of PD-1-positive tumor-infiltrating lymphocytes and PD-L1 expression in IDH-wild-type gliomas than IDH-mutant gliomas. Liu et al. (2020) demonstrated higher CTLA-4 expression in higher-grade IDH-wild-type tumors than lower-grade IDH-mutant tumors. We would summarize these phenotypes as “hot” tumor microenvironments. “Hot” tumors and high expression of PD-L1 were known as hallmarks of sensitivity to immunotherapy (Xiong et al., 2018; Gao et al., 2022), while inexplicably, the efficiency of immunotherapy was limited in gliomas, regardless of the IDH1 status and tumor grades (Blumenthal et al., 2016; Bouffet et al., 2016; Johanns et al., 2016; Lukas et al., 2018; Reardon et al., 2020). We hypothesized that the patient’s inflammatory immune microenvironment is accompanied by other malignant phenotypes.

We then established a 32-gene functional protein association network for NOG and IGFBP2 on the STRING website. These 32 genes were analyzed for gene function enrichment using the KEGG database. The TGF-β signaling pathway, Hippo signaling pathway, PI3K-AKT–mTOR pathway, and signaling pathways regulating pluripotency of stem cells were found to be closely linked to the risk model. It had been reported that stem cell properties could be acquired by tumor cells through EMT. Induction of EMT in immortalized human mammary epithelial cells was sufficient to induce the expression of stem cell markers, enhance self-renewal, and increase the number of tumor-initiating cells (Mani et al., 2008; Shibue and Weinberg, 2017). TGF-β had been thought to be the most important factor inducing EMT *via* the classic Smad and non-Smad pathways (Lamouille et al., 2014; Hao et al., 2019). The Hippo pathway and PI3K-AKT–mTOR pathway had also been proved to be related to EMT (Cordenonsi et al., 2011; Song et al., 2014). It had been reported that there exists a strong correlation between EMT and immune activation. Further analysis demonstrated high expression of immune checkpoints and other druggable immune targets such as PD-1, PD-L1, CTLA-4, OX40L, and PD-L2 in patients with the EMT phenotype (Mak et al., 2016). Therefore, we speculated that the inflammatory immune microenvironment of gliomas is accompanied by the EMT phenotype.

In subsequent analyses, we found that EMT enrichment scores were significantly positively correlated with risk scores, M2 macrophage infiltration, Tregs, PD-L1, and CTLA-4 expression and negatively correlated with CD8^+^ T-cell infiltration. Although Mak et al. (2016) discovered that the EMT phenotype is always accompanied with immune activation and higher expression of immune checkpoint molecules and declared that immunotherapy might have potential, the reality was far from that (10). The inflammatory immune microenvironment is accompanied by EMT, which in turn induces immunosuppression against the inflammatory immune microenvironment. Reversing the EMT phenotype might be necessary for immunotherapy treatment.

Dongre and Weinberg (2019) reviewed the main signaling pathways that induce EMT, including the TGF-β signaling pathway, the WNT signaling pathway, the Notch signaling pathway, the PI3K signaling pathway, and the RAS signaling pathway. Through the enrichment analysis of the aforementioned pathway, we found that the TGF-β signaling pathway is the most important. TGFB1/TGFBR1, TGFB2, and TGFB3 were elected for further investigation. Among these four molecules, TGFBR1 not only had the highest correlation with EMT enrichment scores but also was an independent prognostic factor relative to PD-L1. Immunocorrelation analysis showed that TGFB1/TGFBR1 had more powerful immunosuppressive properties than PD-L1 and CTLA-4, especially in inducing M2 macrophage infiltration and CD8^+^ T-cell depletion. According to the different expressions of TGFBR1 and PD-L1, we performed survival analysis in groups, and the results showed that patients with both TGFBR1 and PD-L1 expression had obvious survival advantages, and the high expression of either molecule led to poor prognosis. This indicates that the combined inhibition of TGFB1/TGFBR1 and PD-1/PD-L1 has a good clinical application prospect.

Through single-cell analysis, we further determined that TGFB1 and TGFBI are mainly derived from microglia and M2 macrophages. As resident macrophages of the central nervous system (CNS), microglia are associated with diverse functions essential to the developing and adult brain during homeostasis and disease (Borst et al., 2021). Microglia-derived TAM (tumor-associated macrophages) increased angiogenesis and suppressed T-cell proliferation. Depletion of TAM provides survival advantages and delays recurrence when combined with standard-of-care treatment such as irradiation (Akkari et al., 2020). Numerous studies have demonstrated that gliomas are infiltrated by immune cells that make up to 30% of a tumor’s mass (Nduom et al., 2015). The predominant population consists of glioma-associated microglia and macrophages, and their numbers inversely correlate with patients’ survival (Gieryng et al., 2017). We speculated that by synthesizing and secreting TGFB1, microglia and M2 macrophages simultaneously induced EMT phenotype and immunosuppression.

Finally, we explored the relative drug sensitivity of the TGFB1 inhibitor in glioma cell lines through the Genomics of Drug Sensitivity in Cancer website and the EMTome website. Although these studies are *in vitro* experiments, the relative sensitivity between different tumor species can still give us some hints that gliomas have relatively good sensitivity relative to most tumors.

In this study, we constructed an immune-related prognostic model associated with the IDH1 mutation status. This model enables further risk stratification of patients with different IDH1 mutation states. By analyzing the immune microenvironment of patients with different risk scores, we found that high-risk patients were more likely to have an inflammatory immune microenvironment and a higher PD-L1 expression level, although clinical studies showed that patients with different IDH1 mutation states did not benefit from PD-1/PD-L1 inhibitors. We speculated that there were other malignant phenotypes that accompanied the inflammatory immune microenvironment, so we performed KEGG analysis on the risk model gene and found that it may be closely related to the EMT phenotype. This hypothesis was confirmed because EMT-related gene sets were significantly enriched in the high-risk group. Subsequently, we found that the EMT phenotype was associated with a decrease in CD8^+^ T cells and an increase in M2 macrophages, which is different from the risk model. By analyzing the main signaling pathways that induce the EMT phenotype, we found that TGF-β was the most important one in gliomas, and TGFB1/TGFBR1 showed stronger immunosuppressive properties than PD-L1 and CTLA-4, especially in inducing an increase in CD8^+^ T cytopenia and M2 macrophages. It is clinically instructive that simultaneous low expression of TGFBR1 and PD-L1 has obvious survival advantages over other expression modes. Through single-cell analysis, we also found that TGFB1 is closely related to microglia and macrophages, especially M2 macrophages, which can explain why the increase in TGFB1/TGFBR1 expression is accompanied by a significant increase in M2 macrophages. Finally, we discussed the sensitivity of TGFB1 inhibitors in gliomas using cell line susceptibility data. From these analyses, we demonstrated a viable clinical strategy in combination with TGFB1/TGFBR1 inhibitors and PD-1/PD-L1 inhibitors for the treatment of high-risk gliomas.

## Data Availability

The original contributions presented in the study are included in the article/Supplementary Material; further inquiries can be directed to the corresponding author.
