# Supplementary material for: An immune and epithelial–mesenchymal transition-related risk model and immunotherapy strategy for grade II and III gliomas
Source: Front Genet. 2023 Jan 4;13:1070630. doi: 10.3389/fgene.2022.1070630 (PMC9909968; doi:10.3389/fgene.2022.1070630)
Supplement: Supplementary file 1 [file Table1.DOCX]

Table S1. OS was compared between groups with different expression of TGFBR1 and PD-L1

| **Group** | | **HR** | **p value** |
| --- | --- | --- | --- |
| TGFBR1_H+CD274_L | TGFBR1_H+CD274_H | 0.48, 95%CI (0.27,0.82) | 6.40E-03 |
| TGFBR1_L+CD274_H | TGFBR1_H+CD274_H | 0.52, 95%CI (0.3,0.91) | 0.02 |
| TGFBR1_L+CD274_L | TGFBR1_H+CD274_H | 0.21, 95%CI (0.13,0.33) | 3.20E-13 |
| TGFBR1_L+CD274_H | TGFBR1_H+CD274_L | 1.19, 95%CI (0.65,2.17) | 0.58 |
| TGFBR1_L+CD274_L | TGFBR1_H+CD274_L | 0.44, 95%CI (0.26,0.76) | 2.10E-03 |
| TGFBR1_L+CD274_L | TGFBR1_L+CD274_H | 0.38, 95%CI (0.22,0.65) | 1.90E-04 |

Notes: log-rank test were applied between groups, P<0.05 was considered statistically significant.
