# Supplementary figures and images for: An immune and epithelial–mesenchymal transition-related risk model and immunotherapy strategy for grade II and III gliomas
Source: Front Genet. 2023 Jan 4;13:1070630. doi: 10.3389/fgene.2022.1070630 (PMC9909968; doi:10.3389/fgene.2022.1070630)

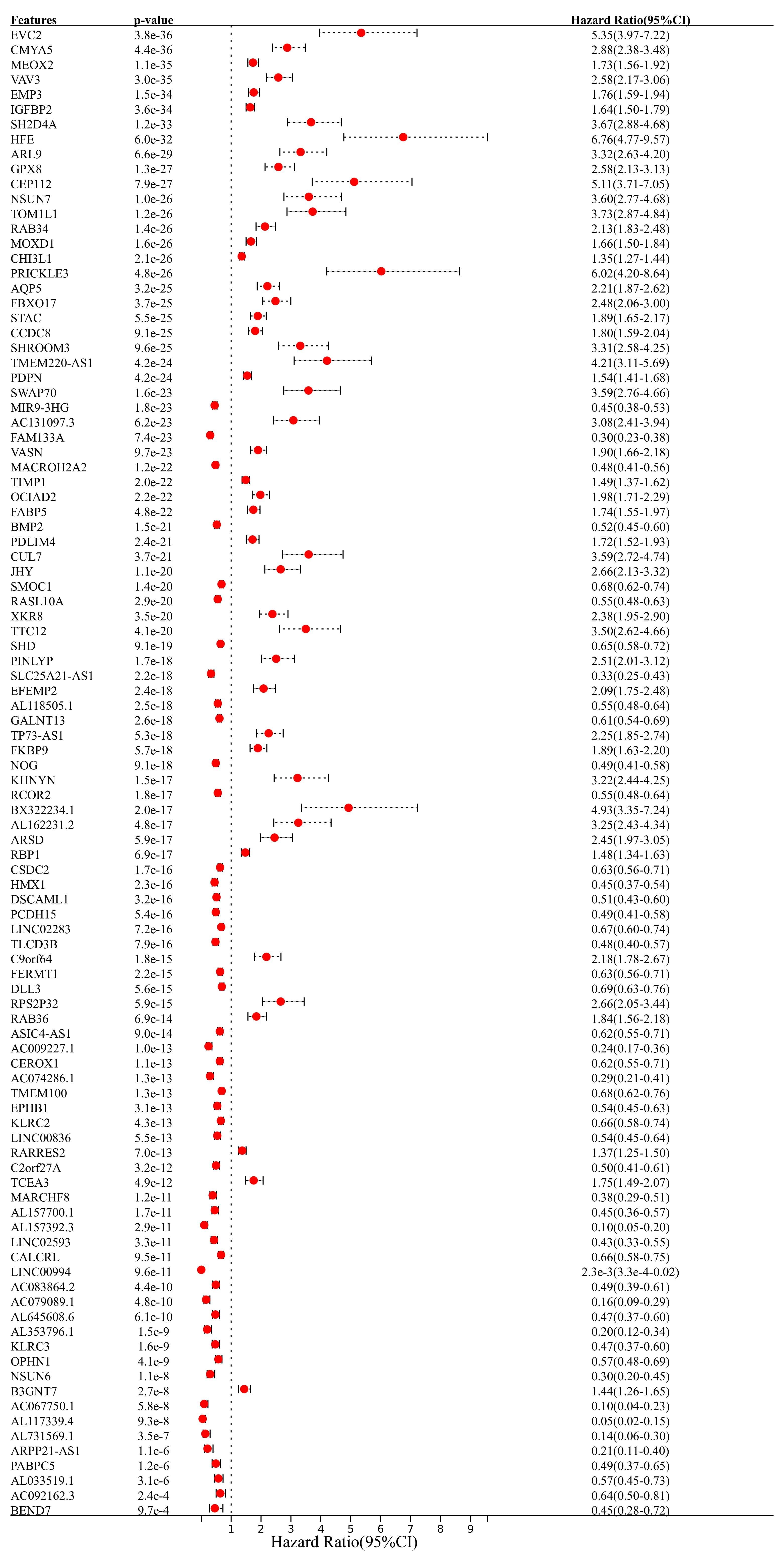

Supplement: Supplementary file 2 [file Image1.JPEG]
